# Applications of Compounds from Coffee Processing By-Products

**DOI:** 10.3390/biom10091219

**Published:** 2020-08-21

**Authors:** Amaia Iriondo-DeHond, Maite Iriondo-DeHond, María Dolores del Castillo

**Affiliations:** 1 Food Bioscience Group, Department of Bioactivity and Food Analysis, Instituto de Investigación en Ciencias de la Alimentación (CIAL) (CSIC-UAM), Calle Nicolás Cabrera, 9, 28049 Madrid, Spain; amaia.iriondo@csic.es; 2 Food Quality Group, Department of Agricultural and Food Research, Instituto Madrileño de Investigación y Desarrollo Rural, Agrario y Alimentario (IMIDRA), N-II km 38, 28800 Alcalá de Henares, Spain; maite.iriondo@madrid.org

**Keywords:** coffee by-products, food applications, health-promoting compounds, market products, nutrients, safety, sustainability

## Abstract

To obtain the coffee beverage, approximately 90% of the edible parts of the coffee cherry are discarded as agricultural waste or by-products (cascara or husk, parchment, mucilage, silverskin and spent coffee grounds). These by-products are a potential source of nutrients and non-nutrient health-promoting compounds, which can be used as a whole ingredient or as an enriched extract of a specific compound. The chemical composition of by-products also determines food safety of the novel ingredients. To ensure the food safety of coffee by-products to be used as novel ingredients for the general consumer population, pesticides, mycotoxins, acrylamide and gluten must be analyzed. According with the priorities proposed by the Food Agriculture Organization of the United Nations (FAO) to maximize the benefit for the environment, society and economy, food waste generation should be avoided in the first place. In this context, the valorization of food waste can be carried out through an integrated bio-refinery approach to produce nutrients and bioactive molecules for pharmaceutical, cosmetic, food and non-food applications. The present research is an updated literature review of the definition of coffee by-products, their composition, safety and those food applications which have been proposed or made commercially available to date based on their chemical composition.

## 1. Introduction

**Figure 1:**
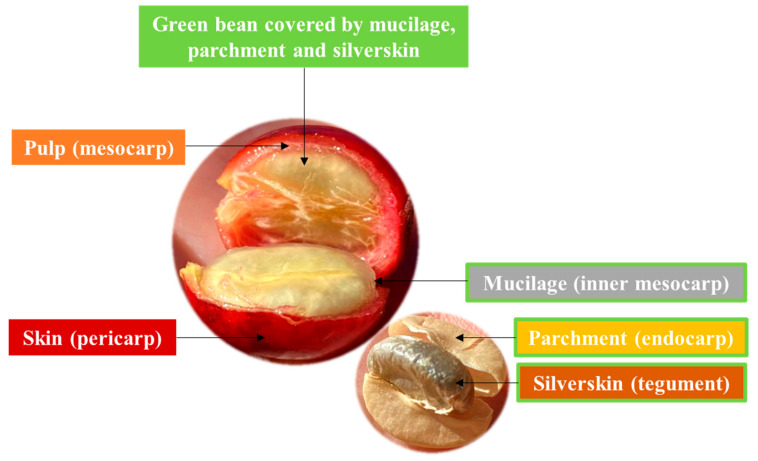
Identification of coffee by-products in the coffee cherry anatomy.

The coffee cherry consists of an outer skin, usually green in unripe and red in ripe fruits, that covers a soft and sweet pulp (Figure 1). This is followed by a viscous and highly hydrated layer of mucilage (pectin layer), a thin yellowish endocarp, the parchment and, finally, the silverskin that covers each hemisphere of the green coffee bean [1].

**Figure 2:**
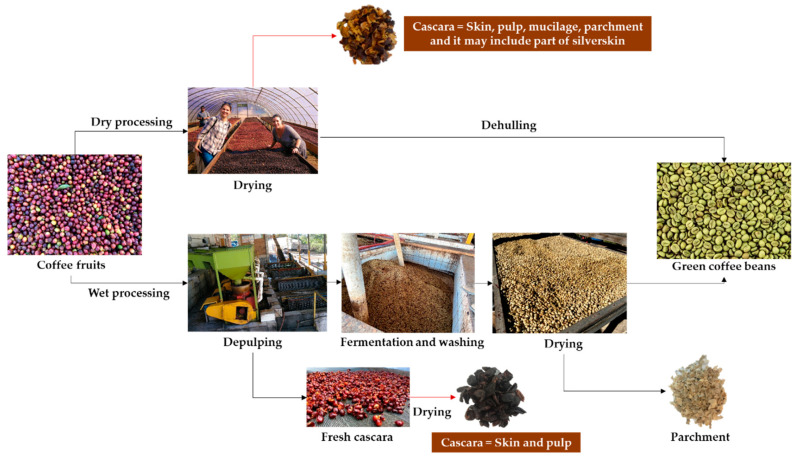
Dry (**top**) and wet (**bottom**) processing of coffee cherries. Photographs were taken in Combrifol and Café Orgánico Marcala S.A. (COMSA), Marcala, Honduras.

The first by-product generated during coffee processing is cascara. The definition and composition of this by-product depends on the type of processing employed: the wet or dry method (Figure 2) [2]. The dry method, commonly used in Robusta producing countries, is technologically simpler. The freshly harvested cherries are spread evenly and dried for 2–4 weeks under the sun until moisture is below 12%. Then, the cherries are mechanically de-husked and the skin, pulp, mucilage, parchment and part of the silverskin (Figure 1) are removed from the beans (Figure 2) [3,4]. Coffee cascara obtained from the dry method comprises nearly 45% of the coffee cherry [5].

The wet processing is commonly used for Arabica, which is commercially more appreciated and requires the use of different instruments to obtain the green bean. First, the skin and pulp covering the beans are removed by a depulper [4]. In Colombia, one of the first producers of Arabica coffee [6], in 100 kg of mature coffee cherries, 39 kg corresponds to skin and pulp [7]. Then, the mucilage is eliminated by fermentation for 24 to 72 h [3] and from 100 kg of fresh coffee cherries, 22 kg of mucilage is generated [7]. Next, the bean covered by the parchment is washed, drained and dried until moisture reaches around 10% [4]. Finally, the parchment is removed using hulling machinery [1]. In Colombia, 39 kg of parchment is generated during processing from 100 kg of fresh coffee cherries [7].

By-products generated until the obtainment of the green coffee bean are generated in coffee producing countries. Green beans are then exported to coffee-consuming countries and stored until roasting [2]. During the roasting process, the silverskin (CS, the thin tegument that covers the bean) is detached and represents the only by-product of the coffee roasting industry [3]. In Spain, the roasting of 120 kg of coffee generates 2.5 kg of CS (Supracafé S.A., *pers. comm.*). Finally, roasted beans are ground, and the coffee beverage is prepared at home or processed for soluble/instant coffee leading to the generation of the last coffee by-product, spent coffee grounds (SCGs) [1]. About 2 kg of wet SCGs are obtained for each kilogram of soluble coffee produced [2]. Table 1 shows the origin, type of processing, processing step and the nutritional and bioactive compound composition of all coffee by-products.

**Figure 3:**
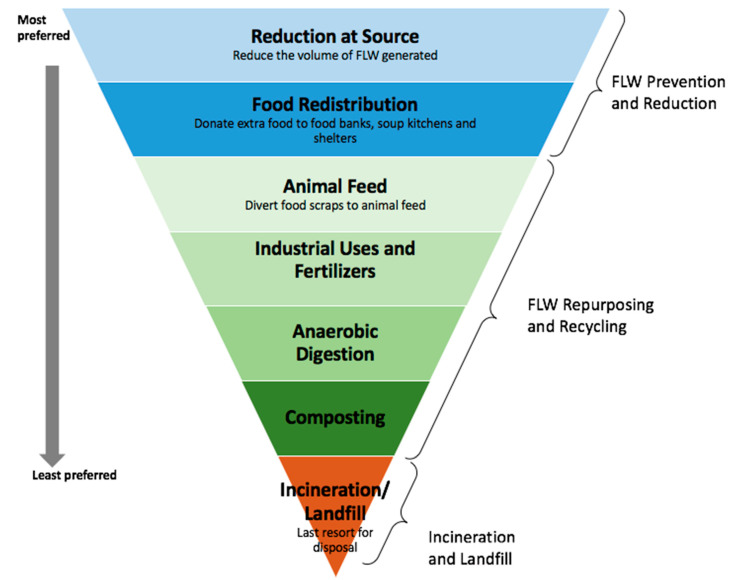
The food hierarchy proposed by the Food and Agriculture Organization of the United Nations (FAO, 2019). FLW, Food losses and waste.

To increase the eco-sustainability of the coffee industry, by-products should be exploited before they become waste. Dealing with food waste is of great importance to combat hunger, raise income and improve food security in the world’s poorest countries [8]. To maximize the benefit for the environment, society and economy, the Food Agriculture Organization of the United Nations (FAO) has developed a Code of Conduct (CoC) for the reduction in food loss and waste and has proposed an inverted pyramid setting priorities on how to best reduce food waste and save natural resources (Figure 3) [9].

Their first recommendation is to avoid generating waste in the first place. Therefore, the present research is an updated literature review of the nutritional value and health promoting properties of coffee by-products, their safety and those food applications that have been proposed or made commercially available to date based on their chemical composition. High-value components such as proteins, polysaccharides, fibers, flavor compounds and phytochemicals can be extracted and re-used as novel food ingredients [10]. In the end, the aim is to re-use the whole by-product to avoid generating any more waste.

## 2. Materials and Methods

The present narrative review was conducted by a literature search consulting the Google Scholar, Web of Science and Scopus databases. Search terms related to coffee by-products (“coffee by-products”, “coffee cascara”, “coffee mucilage”, “coffee parchment”, “coffee silverskin”, “spent coffee grounds”), were combined with the following search terms: “nutritional value”, “health-promoting properties”, “safety” and “applications”. Data on legislation were consulted from the United Nations Food and Agriculture Organization (FAO) and the European Food Safety Authority (EFSA). In addition, commercially available applications were found by searching in Google. The selection of the papers to be included in the review was performed after a thorough study of their content by the authors. The selection process resulted in the identification of 130 eligible research articles and webpages.

## 3. Nutritional Value and Health Promoting Properties

Coffee by-products have been proposed as a potential sustainable source of macro-, micro- and non-nutrient bioactive compounds. Table 2 summarizes the nutrition claims that can be attributed to each coffee by-product based on their nutritional composition and their health-promoting properties. Despite the fact that with the data taken from the literature, certain nutritional claims are not reached, depending on the composition of each specific sample, a particular by-product could reach a certain nutritional claim. For instance, in some cases cascara, spent coffee grounds and the ingredients obtained from them may be considered as source of proteins. This macronutrient is the second most abundant component found in both raw materials (Table 1).

Coffee cascara is enriched in anthocyanins, which contain powerful inhibitors of α-glucosidase and α-amylase enzymes. As these enzymes play an important role in the management of glucose metabolism, the use of anthocyanin extracts from coffee cascara has been proposed as an antidiabetic agent to improve postprandial blood glucose metabolism [29]. These anti-diabetic properties have also been studied in novel gluten-free breads and yogurts containing cascara extract that showed inhibition against α-glucosidase after in vitro digestion [28,54]. On the other hand, anti-inflammatory properties of coffee cascara obtained from a semi-dry processing were demonstrated by the inhibition of the release of the pro-inflammatory cytokine IL-8 on gastric epithelial cells when inflammation was induced by TNF-α [32]. Caffeine present in coffee cascara may contribute to the overall anti-inflammatory effect observed in this by-product [55,56]. A coffee cascara aqueous extract [57], contained 1.4% caffeine, 0.2% chlorogenic acids (CGA) and presented antioxidant capacity in vitro (80.6 mg eq. CGA/g). Antioxidant properties of this extract at 1 mg/mL were also confirmed in human liver cells (HepG2) [24]. This extract may be a potential agent for the prevention of cellular damage induced by oxidative stress.

With regard to coffee parchment, it presents in vitro hypoglycemic properties, showing high glucose adsorption capacity (50–200 mmol/L) and α-amylase inhibition (52%), as well as in vitro hypolipidemic properties by pancreatic lipase inhibition (43%) and cholesterol binding (16.6 mg/g) [19]. Aguilera et al. (2019) carried out an aqueous heat-assisted extraction to obtain an extract enriched in phenolic compounds from coffee parchment. Their results demonstrated the need to mill parchment to promote the extractability of phenolic compounds, such as CGA, vanillic acid, protocatechuic acid and *p*-coumaric acid [57].

The anti-obesity effect of coffee may be attributed to caffeine [58] and CGA [59] that are also present in CS. The anti-obesity properties of CS have been studied in novel antioxidant beverages based on CS from Arabica and Robusta species to determine their inhibitory effect on in vivo fat accumulation using *Caenorhabditis elegans* as an animal model [36]. Both beverages reduced body fat by 21% and 24%, respectively, possibly due to the presence of these compounds at physiologically active doses [36]. The health-promoting properties of CS may also be attributed to melanoidins present in this by-product [35,36]. Several biological activities, such as antioxidant, antimicrobial, anticariogenic, anti-inflammatory, antihypertensive and antiglycative activities, have been attributed to coffee melanoidins [60]. Phenolic compounds found in aqueous extracts from coffee cascara and CS also alleviated the complications of adipogenesis and inflammation in vitro [33].

In an in vivo study with Wistar rats, a CS aqueous extract (CSE) reduced total cholesterol and triglycerides plasma levels after a 45-day treatment with CSE (2.2 and 0.8 mg caffeine and CGA/kg body weight (b.w.)). Furthermore, CSE reduced pancreatic lipase activity in vitro (41.73%) [37]. This could explain the mechanism of action of the observed reduction in total cholesterol and triglycerides since pancreatic lipase is a key enzyme in fat digestion. Together, these results support the liporegulatory character of CS through pancreatic lipase inhibition, and therefore, its preventive and therapeutic effect in obesity.

The components present in CSE also have a positive effect on pancreas health, thereby reducing the risk of diabetes. This by-product produces increased glucose tolerance [37], enhances insulin sensitivity and secretion [37,39], inhibits enzymatic α-glucosidase activity [39], inhibits advanced glycation end products (AGEs) formation through the interaction of CGA and its derivatives with protein backbone [22,61,62], and protects against oxidative stress [63]. Moreover, CSE protects pancreatic tissue in vitro against oxidative stress induced by the commonly used diabetogenic agent streptozotocin (STZ) [64]. As these effects have an impact on diabetes development, CSE may be useful in both the prevention and treatment of diabetes.

The antioxidant capacity of CSE has been proved at a genomic level [65]. CSE protected against benzo(a)pyrene (B(a)P) induced DNA damage, strand breaks and oxidized purines and pyrimidines in HepG2 cells. This extract had a higher protective effect against B(a)P-induced oxidative DNA damage compared to that observed for the strand breaks, possibly due to its well-known antioxidant capacity. Free CGA linked to other chemical structures seemed to contribute to the observed chemoprotective effect of CSE.

The high dietary fiber content of CS (up to 55%), predominantly insoluble fiber, can potentially benefit the intestine and gut microbiota [23,25,41]. CS preferentially supports bifidobacteria growth in vitro, suggesting that its consumption may have some prebiotic effects [35]. Studies carried out by our research group have shown the fiber fermentability of CSE in vivo [24]. Total short chain fatty acids (SCFAs) significantly increased (*p* < 0.05) in feces of male Wistar rats treated with CSE (1 g/kg b.w.) for 28 days compared to male control animals. In addition, an isolated melanoidin fraction (MEL) obtained from CS, mainly composed of dietary fiber (75%) and melanoidins (15%), showed antioxidant capacity in vitro and was effective against induced oxidative stress in rat intestinal cell lines. Furthermore, the fiber effect of this fraction was confirmed in vivo when rats treated with MEL for 28 days showed an acceleration of intestinal transit compared to control animals [66].

Previous studies have shown that SCGs also have a positive effect on beneficial bacteria, increasing the number of lactobacilli and bifidobacteria, therefore being a good source of prebiotic compounds [25]. SCGs’ dietary fiber can be fermented by colon microbiota, producing SCFAs with anti-inflammatory properties, such as inhibition of nitric oxide production and other inflammatory mediators such as IL-10, CCL-17, CXCL9, IL-1β and IL-5 cytokines [51]. Consequently, SCGs have been proposed as a protective agent against chronic inflammatory diseases, such as inflammatory bowel disease and rheumatoid arthritis. In addition, dietary fiber from SCGs has recently been reported to stimulate the release of the satiety hormone, serotonin, and glucagon-like peptide-1 [67]. The antioxidant properties of the dietary fiber of SCGs have mainly been ascribed to the presence of phenolic compounds present in their polymeric structure [49].

In vivo studies carried out in rats treated with SCGs for 28 days showed a significantly lower (*p* < 0.1) accumulation of lipids in the liver and a higher excretion of them in feces in rats treated with SCGs compared to non-treated animals [68]. In addition, SCGs acutely accelerated intestinal motility in rats. Therefore, SCGs might be considered a sustainable, safe and healthy food ingredient with a potential for preventing hepatic steatosis due to their effect as dietary fiber with a high fat-holding capacity. Studies suggest that insoluble fiber may play an important role in weight loss in high-fat diets by adhering fat to its complex structure, and it has been inversely associated with the risk of type 2 diabetes [69,70].

## 4. Safety and Regulatory Status

To ensure the protection of human health in Europe, specific legislation (Regulation (EU) 2015/2283) [71] must be applied to food waste and by-products before they can be used as novel food ingredients. According to this regulation, “a food is considered to be novel if it has not been regularly consumed by humans in the EU before 15 May 1997, when the first novel food Regulation came into force” [71]. Information regarding the analysis of toxic substances and toxicological studies should be included in the application for the authorization of a novel food according to Regulation (EU) 2015/2283 [71]. The main consideration regarding the commercial use of coffee by-products as food ingredients is their regulatory status. Updated information on coffee by-products’ novel food status considering regulation (EU) No 2015/2283 can be found in a review recently published by Klingel et al. [72].

Limitations on the use of coffee by-products are connected to their caffeine content. Coffee by-products have different caffeine levels, but in all cases their caffeine content is lower than that found in green and roasted coffee beans [73]. Although further studies should be conducted in this field, results published to date suggest that caffeine content does not need to be considered a safety concern in the application of coffee by-products as food ingredients. The caffeine content in foods formulated with coffee by-products should be below the European Food Safety Authority (EFSA) safety level for daily caffeine consumption of 400 mg for the general population and 200 mg for lactating women. For children and adolescents, the available information is insufficient to derive a safe level of caffeine intake [74]. Therefore, no limitations on the use of these by-products as food ingredients for human nutrition need to be considered.

A limitation on the use of CS and SCGs, obtained after the roasting process, is acrylamide, a processing contaminant. Acrylamide is formed during the roasting of coffee beans through the Maillard reaction, during which the aroma and the color of coffee beans are also produced. This reaction takes place when precursors, for example, reducing sugars, such as glucose and fructose, and asparagine, are present in raw materials in combination with a high temperature and a long cooking time [75]. Low levels of 346 and 37 µg/kg of acrylamide have previously been found in CS and SCGs, respectively [49,76]. These values are under the limit established by the EFSA for coffee and instant coffee, 450 and 900 µg/kg, respectively [77]. Traces of acrylamide (223 μg/kg) have been recently found in an aqueous extract of coffee cascara [14]. Thus, it is of great importance to search for alternative drying methods to mitigate the appearance of this undesirable compound and to ensure the food safety of this by-product.

Our research group has recently confirmed the safety of coffee cascara, parchment, CS and SCGs by the absence of pesticides and mycotoxins (aflatoxin B1 and enniantin B) [42,68]. Ochratoxin A (OTA) values reported in all by-products were below the limit established by the European Commission (5 μg/kg) [78]. OTA is a mycotoxin produced by *Aspergillus ochraceus* and *Penicillium verrucosum* that tends to bioaccumulate along the food chain. This mycotoxin can induce renal toxicity, nephropathy, and immunosuppression [79]. Coffee is considered a secondary source of OTA in the human diet. OTA is already present in coffee before storage, as contamination can occur due to climatic conditions, coffee fruits falling onto the soil, transportation, and so on. Therefore, the critical steps leading to the accumulation of this mycotoxin are the harvesting and postharvest handling of coffee cherries [40]. Other reported values of OTA in CS were between 18.7 and 34.4 μg/kg, which is about three times higher than the European Commission limit for coffee products (5 μg/kg) [79]. However, Ferraz et al. (2010) demonstrated that OTA can be destroyed during roasting [80]. Even when the coffee beverage is prepared from highly contaminated green beans, the coffee-transforming process is able to reduce the amount of OTA that represents a risk to human health [40]. However, the type of preparation of the beverage affects the OTA content, which is sometimes higher than that expected from roasted coffee [81]. Hot water extraction of roasted coffee containing OTA during a long time (such as filtered coffee) showed higher content of OTA compared to cold-water extraction or to espresso coffee [81]. Although roasting can reduce OTA content, there is still a toxicological problem. The effect of coffee roasting on its OTA content is a controversial subject. Therefore, rigorous quality controls should be established along the coffee-bean processing chain to reduce OTA and ensure the safety of green coffee beans and coffee by-products [40].

In addition to determining biological, chemical and processing contaminants, the safety of coffee by-products needs to be further confirmed by toxicity studies. The safety of CoffeeBerry^®^ products (a powdered extract, a water extract and a water–ethanol extract) derived from the whole fruit has been evaluated in genotoxicity studies, short-term oral toxicity studies and a 90-day dietary toxicity study [82]. None of the coffee cherry extracts showed mutagenic or genotoxic potential, and in short-term and 90-day toxicity studies, no adverse effects were observed at the studied concentrations [82].

Our research group has completed the safety evaluation of coffee by-products by an acute toxicity study, which showed no visible signs of toxicity, abnormal behavior or mortality after the administration of 2 g/kg b.w. of cascara aqueous extract, parchment, CSE and SCGs to female Wistar rats [42,68]. Further toxicological studies have been carried out for CSE. First, the absence of genotoxicity and cytotoxicity, which is basic in chemical risk assessment [71], was confirmed in human liver cells (Hep G2) treated with different concentrations of CSE [65].

Regarding additional in vivo toxicity studies, the repeated oral administration of CSE at a dose of 1 g/kg for a period of 28 days was not toxic to male and female Wistar rats [24]. On the other hand, the repeated intake of SCGs at a dose of 1 g/kg b.w. for 28 days was not noxious to the animals [68]. To the best of our knowledge, these are the only in vivo studies regarding the toxicological effect of coffee by-products. Toxicological data derived from the investigation carried out by our research group allowed the request of the Novel Food Application to the EFSA (by Bionok Healthcare S.L.) for the use of coffee silverskin extract as a food ingredient.

## 5. Food Applications of Coffee Processing By-Products

### 5.1. Research Proposals

#### 5.1.1. Cascara

Coffee cascara is currently used for composting in coffee-producing countries. Other applications proposed for this by-product are production of biofuel [83], enzymes [84], biosorbents [85], particleboard [20] and animal feed [86]. In the food industry, different studies propose cascara for its use as a source of anthocyanins and dietary fiber, among other applications [87]. In 2015, Ramirez Velez and Jaramillo Lopez patented the extraction of honey or flour from coffee cascara for its use in products for human or animal consumption, drugs and cosmetics or as a raw material for the production of alcohol for fuel (ethanol) [88]. On the other hand, dry coffee cascara has also been proposed for its use in the development of high-fiber salty cookies [89]. These cookies were enriched with phenolic compounds that possessed antioxidant capacity.

**Figure 4:**
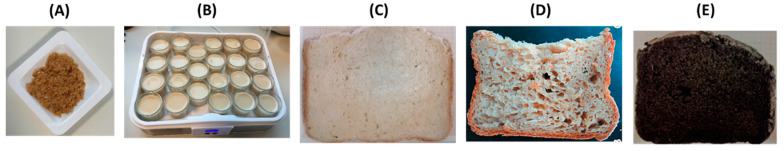
(**A**) Powdered Instant Cascara. (**B**) Coffee cascara extract and inulin yogurts. (**C**) Wheat bread slice. (**D**) Gluten-free bread containing cascara extract. (**E**) Gluten-free bread enriched in cascara dietary fiber. Novel foods were developed by the Food Bioscience Research Group at the Instituto de Investigación en Ciencias de la Alimentación (CIAL, UAM-CSIC), Madrid, Spain.

In order to increase the number of applications for this by-product and the diversification of the coffee sector, our research group proposes drying coffee cascara and carrying out a simple fractionation process by aqueous extraction [42]. The aqueous extract derived from this process, enriched in phytochemicals, might be used for technological and health purposes. On the one hand, it could be used as a food preservative or as a food colorant. Coffee cascara obtained from wet coffee processing has been proposed as a potential source of anthocyanins for natural food colorants. In the wet process, coffee cascara is removed before drying and its color is rapidly degraded by the action of enzymes or oxygen. As a result, large amounts of natural colorants are wasted [90]. Cyanidin 3-rutinoside was characterized as the most abundant anthocyanin, responsible for the red color observed in the outside of the fresh coffee berry [29]. Prata and Oliveira (2007) carried out the extraction of monomeric and polymeric anthocyanins in five varieties of coffee cascara and the average content of monomeric anthocyanins in cascara was 19.2 mg of pigment per 100 g of fresh cascara [91]. This by-product should be appropriately managed in the drying process to preserve these compounds. Coffee cascara possesses great potential as an economic source of natural colorants. For instance, coffee cascara extract has been used in the development of gluten-free breads to provide the typical appearance of wholemeal bread [54] (Figure 4C).

Another proposed application for this cascara extract is in the development of healthy and sustainable yogurts (Figure 4B). Cascara extract was added to yogurts for its α-glucosidase inhibition properties, which may affect the regulation of carbohydrate metabolism and enhance gut satiety signals [28]. In this study, a novel yogurt formulation containing a combination of coffee-cascara extract (10 mg/mL) and 3% inulin that is well-tolerated and sensory-accepted with the nutritional claim “source of dietary fiber” was obtained (Figure 4).

Our research group has developed an “instant cascara” beverage based on the cascara powdered aqueous extract (Figure 4A). A safe instant beverage with antioxidant properties was obtained, to which the following nutrition claims can be assigned: “low fat”, “low sugar” and “source of potassium and vitamin C” [14]. Photosensitivity and heat resistance studies seemed to indicate that instant beverages in solution are more susceptible to color changes by light and temperature (40 °C) exposure. It is therefore suggested a package that protects the product from light is used and the product is stored in a cool, dry place.

The extraction waste, the insoluble fraction obtained after the aqueous extraction of coffee cascara can also be repurposed in the food industry sector. This insoluble fraction is also generated during the elaboration of the commercially available “cascara” beverages [92]. Recent studies carried out by our research group have used this isolated coffee cascara dietary fiber in gluten-free breads to improve their nutritional and sensory quality [93]. The addition of the isolated coffee cascara dietary fiber (3% and 4.5%) allowed increasing dough yield, less crumb firmness and a higher crumb elasticity. In this investigation, a certificated gluten-free bread with improved nutritional (“source of protein and high in dietary fiber”) and physicochemical properties and a good sensorial profile was obtained (Figure 4D).

#### 5.1.2. Mucilage

According to the International Coffee Organization, coffee mucilage could be used in foods as an unrefined source of pectin, antioxidants and flavonoids. All these compounds have raised special interest in the food industry [94]. Coffee mucilage has also been proposed for its use as a honey for human consumption [88]. The chemical composition of this honey showed 30–40% moisture, 55 °Bx, 4% proteins, 2% fiber and a polyphenol content corresponding to 380 mg gallic acid equivalents/100 g. This coffee honey with a high sugar content was obtained by means of a vacuum dehydration step at a temperature below 65 °C, obtaining a product with minimum nutritional damage by heat, and high digestibility and palatability [88].

#### 5.1.3. Parchment

A recent study proposes parchment as a promising low-calorie functional ingredient for dietary fiber enrichment in foods with hypoglycemic and hypolipidemic properties [19]. Results from this research showed that coffee parchment flakes possessed higher oil-holding, water-holding, absorption and swelling capacities than parchment flour [19]. Previous preliminary studies carried out by our research group have evaluated the potential of coffee parchment fiber in gluten-free bread formulations. Enriched breads contained 6.25% parchment flour. The final gluten-free product had the nutrition claim “high in fiber” thanks to the supplementation with the coffee by-product flour and was accepted by consumers [95]. In addition, Cubero-Castillo et al. (2017) developed a cookie with 2% coffee parchment flour as a dietary fiber source and with antioxidant capacity. In this study, coffee parchment flour did not generate rejection in consumers, which is a positive characteristic since dietary fiber from cereals generally has a low acceptance [96].

Considering that cellulose and lignin are the major components in parchment, the best use of this by-product seems to be as a source of cellulose for other industrial uses. In accordance with the patent by Joseph Apuzzo, our research group proposes the use of parchment as a source of cellulose for the production of cellulose-based materials such as coffee filters or food packaging [97]. Sun-dried parchment obtained after the hulling step could be processed to be converted into sustainable materials. Cellulose film packaging material is being increasingly used in personal care, food and beverage industries [98]. Lignocellulosic fibers and lignin are two of the most important natural bioresources in the world and show great potential to improve biodegradability by replacing synthetic fibers in bioplastics. Lignin has shown the potential to function as a plasticizer, stabilizer or bio-compatibilizer in bioplastics, making parchment a good candidate to be used in sustainable packaging [99].

#### 5.1.4. Silverskin

Fiber-enriched foods have been developed in recent years to increase dietary fiber consumption to reduce the risk of chronic diseases [100]. CS has been used as dietary fiber to formulate breads, as it reduces caloric density and increases dietary fiber content [101]. CS has also been used as a colorant and dietary fiber source, to achieve a healthier, nutritious and a high-sensorial-quality biscuit. CS improved the moisture, texture, thickness and color of the novel biscuits [76]. Furthermore, cakes were formulated with up to 30% water-treated CS as a flour substitute [100,102], enhancing moisture content, textural and sensory attributes. Cakes with water-treated CS presented similar physical and sensory characteristics to the control cake [100].

As for coffee cascara, we propose carrying out an aqueous extraction to CS generated during the roasting process of green coffee beans to generate two different food ingredients for human consumption: an aqueous extract enriched in nutrients and non-nutrient bioactive compounds (CSE) and an insoluble dietary fiber fraction.

**Figure 5:**
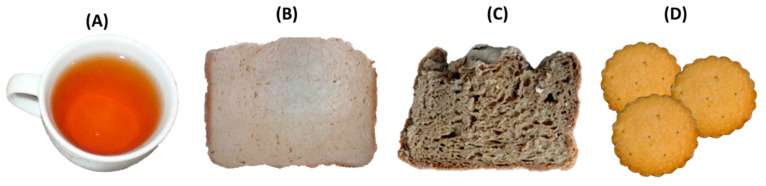
(**A**) Novel beverages from Arabica and Robusta CSE. (**B**) Wheat bread slice. (**C**) Gluten-free bread with CSE. (**D**) Biscuits with CSE. Novel foods were developed by the Food Bioscience Research Group at the Instituto de Investigación en Ciencias de la Alimentación (CIAL, UAM-CSIC), Madrid, Spain.

Based on its nutritional and chemical composition, CSE could be consumed as a food supplement, or it could be added to the most suitable food matrix considering the sensory acceptance of consumers. Very recently, Angeloni et al. studied the volatile fraction of CS for the first time, demonstrating that it contains an interesting odor-active compound fraction with high similarity to coffee beans [103], suggesting exploitation of this by-product in novel food production. In 2013, our research group patented (WO 2013/004873) its use in food and cosmetic applications [45]. Studies regarding food applications of CSE in anti-obesity beverages [36], biscuits [76] and gluten-free breads [54], were previously published (Figure 5).

The insoluble fraction remaining from the aqueous extraction of CS can also be used as a different food ingredient rich in dietary fiber with the final aim of recovering the whole by-product. In previous studies, CS dietary fiber was added to biscuits to improve their nutritional quality [76].

#### 5.1.5. Spent Coffee Grounds

**Figure 6:**
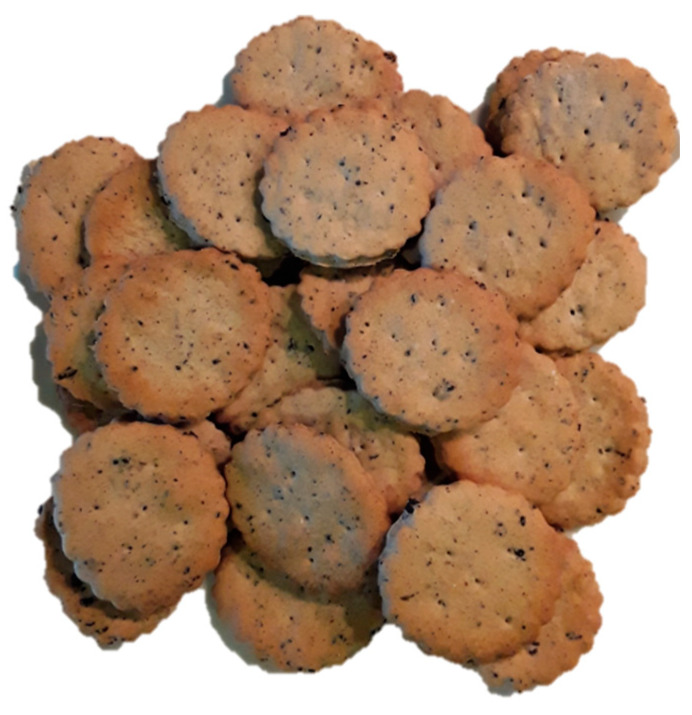
Biscuits containing SCGs developed by the Food Bioscience Research Group at the Instituto de Investigación en Ciencias de la Alimentación (CIAL, UAM-CSIC), Madrid, Spain.

In Europe, most SCGs are currently being incinerated or disposed of in landfills [104]. The economic and environmental costs of disposing of SCGs are undesirable. For this reason, alternative applications for repurposing SCGs are needed [105]. The potential applications for SCGs, from the most to the least preferred option considering the food waste hierarchy proposed by the FAO (Figure 3), that are currently being researched, are as follows:

1. Nutraceuticals. SCGs have been proposed as a protective agent against the onset and of chronic inflammatory diseases, such as inflammatory bowel disease and rheumatoid arthritis. This protective effect is associated to metabolites produced by colonic fermentation of SCGs (SCFAs), which exhibited strong antiinflammatory potential by suppressing nitric oxide production and inhibiting inflammatory mediators such as IL-10, CCL-17, CXCL9, IL-1β, and IL-5 cytokines [51];
2. Food ingredient. Our research group has patented the use of SCGs as a food ingredient rich in antioxidant dietary fiber for bakery products (Figure 6). This ingredient could be directly applied in the manufacture of pastry and confectionery foods such as bread, cookies, and breakfast cereals, among others, making it a simple, low-cost alternative [49,106,107];
3. Novel beverages. A distilled beverage with a coffee aroma was developed by aqueous extraction of aromatic compounds from SCGs, supplemented with sugar and the production of ethanol [108];
4. Food preservative. The addition of SCGs to meat and other foods were shown to provide antioxidant properties inhibiting lipid oxidation, and also antimicrobial properties reducing pathogenic bacterial growth and, therefore, spoiling of food [25];
5. Skincare products. An emulsion containing 35% of oil extracted from SCGs presented promising characteristics as a sunscreen. This formulation is industrial-scalable and suitable for topical use according to the rheological, mechanical and safety assessment [109];
6. Animal feed. SCGs may be used as an alternative to conventional feed ingredients because of their nutrient composition and relatively low cost [110];
7. Biodiesel. This is one of the most popular research topics surrounding SCGs for energy use. It consists of first extracting the oils present in SCGs, and transesterifying them into Fatty Acid Methyl Esters (FAME), commonly referred to as biodiesel [111];
8. Bioethanol. The oil-free SCGs from biodiesel production can be reused as a source of carbohydrates for ethanol production by fermentation [111];
9. Solid biofuel. SCGs can be used alone or mixed with other biomasses such as pine sawdust, and then this mixture can be submitted to pelletization. Pellets produced from SCGs were comparable to other biomass materials, but still had higher particle emissions than alternatives such as pure sawdust [112];
10. Composting and fertilizer. Direct application of SCGs to soils was found to be damaging due to their high C/N ratio, phenol content and acidity. Positive results have been obtained from studies on the effect of mixing SCGs with other organic wastes in different ratios [113];
11. Materials for construction industry. SCGs were mixed with other waste materials such as recycled glass, bagasse ash or fly ash in order to produce materials with high compressive strengths, suitable for use as a subgrade material [114];
12. Bioplastics. Triglycerides extracted from SCGs using a green chemistry approach, based on supercritical CO_2_ extraction, seem to be promising candidates for the production of bioplastics [115];
13. Adsorbent of contaminants. SCGs were proven to be an effective adsorbent for a wide range of contaminants such as metal ions, dyes and bioactive compounds present in water [105].

The nutritional value of SCGs makes them a good candidate for application in the food industry. In fact, using SCGs as a food ingredient would be the most efficient application for this by-product.

### 5.2. Commercially Available Products

#### 5.2.1. Cascara

Nowadays, tea made from coffee cascara has become a popular beverage commercialized by different companies all over the world. This beverage is known as “Cascara Tea” and is sold as a super food with polyphenols with antioxidant properties and with Brain-Derived Neurotrophic Factor (BDNF) that contributes to maintaining the brain’s health [30]. Supracafé, a Spanish coffee company, commercializes coffee cascara tea bags with only cascara and three different flavors: berries, orange and cinnamon, and orange and passion fruit [116]. Other different commercial beverages based on coffee cascara are attracting the interest of Western consumers. Bai Brands is a beverage company founded in 2009 in Princeton, New Jersey, that uses what it calls superfruit extract as an ingredient in its beverages. The term “superfruit” refers to the outside part of the coffee cherry, known as the coffee cascara, which contain antioxidants and caffeine [117]. KonaRed Corporation is a Hawaiian coffee company that also produces beverages from coffee cascara [118]. Another commercial product is Caskai Sparkling Cascara Infusion, which are lightly carbonated non-alcoholic beverages made from our sun-dried cascara [92].

Different companies are already using coffee cascara as a source of dietary fiber. Pectcof B.V. extracts soluble dietary fiber from coffee cascara for both food and non-food applications. Due to its properties as an emulsifier and stabilizer, this extracted coffee dietary fiber is a promising new ingredient for the food and beverage industry [119]. The Coffee Cherry Co. has also developed flour from coffee cascara, and it proposes its application as a food ingredient in different food matrices such as breads, cookies, muffins, squares, brownies, pastas, sauces and beverages. This flour is gluten-free, has five times more fiber than wholegrain wheat flour, contains antioxidants, iron, potassium and proteins, and has a low fat content [120]. Moreover, Modcup sells a cascara chocolate bar that includes coffee cascara to increase levels of antioxidants and natural sweetness in order to reduce the amount of sugar cane used in the process of making chocolate [121].

In addition, the enriched extract obtained from coffee cascara may also be used for healthy purposes. VDF FutureCeuticals Inc., a manufacturer of fruit, vegetable and grain ingredients, has been producing extracts from the coffee cherry for human health for over 10 years [122]. These extracts are proposed to be used to improve human health, specifically brain health, as ready-to-drink beverages, supplements or functional snacks. NeuroFactor™ is a natural, patented extract of whole coffee fruit that contains a unique polyphenol profile that has been shown in a clinical study to stimulate the production of BDNF, a key neuroprotein involved in overall brain health. BDNF has been widely reported to play a critical role in neuronal development, maintenance, repair and protection against neuro-degeneration.

#### 5.2.2. Mucilage

To date, the only application for coffee mucilage is a honey that is sold as a concentrate of antioxidants with health-promoting properties such as strengthening the immune system [34].

#### 5.2.3. Parchment

To the best of our knowledge, there are no commercially available products derived from coffee parchment. Considering its chemical composition, we propose to use this by-product as a sustainable food packaging material in the food industry.

#### 5.2.4. Silverskin and Spent Coffee Grounds

CS and SCGs, which are the most studied by-products, are currently being underutilized and have not been industrially exploited by the food sector. There are no commercially available products containing these potential novel ingredients. It is important for coffee industries to make an effort to valorize CS and SCGs from coffee processing to increase the sustainability of the process, increase economical income and create new jobs in producing countries [11].

In the case of SCGs, non-food applications have led to the commercialization of different products. For instance, the German company Kaffeeform reuses SCGs from six servings of coffee to make a coffee cup and saucer [123]. SCGs are also being commercially used in the production of sustainable yarn for clothing and for the elaboration of coffee-infused recycled polyester socks for odor control [124,125]. A UK-based green energy firm, Bio-bean, recycles SCGs and turns them into “Coffee Logs” as a solid biofuel for multi-fuel stoves or barbecues [126]. Another English company, Greencup, transforms 200–300 tons of SCGs each year into 100% organic fertilizer [127]. Interestingly, Jeweller Rosalie McMillan in the UK creates pieces containing 70% recycled coffee grounds, collected mostly from offices in London [128].

## 6. Conclusions

Based on their nutritional composition, dietary fiber is the main component in all coffee by-products. The dietary fiber present in by-products is of different nature (soluble and insoluble), lacks allergenic proteins and could be easily extracted for use as an ingredient within the concept of a healthy diet. The second main component in coffee by-products is protein, which could also be isolated and employed as an ingredient. Cascara and silverskin are also a potential source of micronutrients, vitamins and minerals, such as ascorbic acid and potassium. The main phenolic compounds found in coffee by-products are chlorogenic acids, with potential health-promoting properties, such as antioxidant, anti-diabetic and anti-obesity.

Studies carried out to date indicate that by adequately controlling food safety in terms of biological contaminants (fungi and mycotoxins) and chemicals (pesticides and acrylamide), coffee by-products could be used as food ingredients due to their interesting content in nutrients and non-nutrient health-promoting compounds. Due to their particular composition and safety, botanical extracts obtained from coffee cascara and silverskin can be employed in nutritional, health and cosmetic applications. The scientific knowledge provided in this publication is very necessary for the certification of these by-products as novel food ingredients, taking into account the EFSA regulations.

## Figures and Tables

**Table 1:** Origin, type of processing, processing step and the nutritional and bioactive compound composition of coffee by-products. Data are expressed in% of dry matter.

By-Product	Cascara	Mucilage	Parchment	Silverskin	Spent Coffee Grounds
	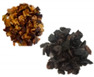	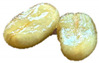	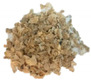	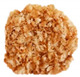	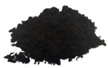
Origin	Producing countries	Producing countries	Producing countries	Worldwide	Worldwide
Type of processing	Dry/wet	Wet	Wet	Dry/wet	Dry/wet
Processing step	Pulping	Fermentation	Hulling	Roasting	Brewing
Kg by-product/100 kg cherry */bean **	39–45 *	22 *	39 *	2.08 **	65 **
**Macronutrients**					
Carbohydrates (%)	45–89	45.8	0.45	44	82
Total fiber (%)	18–32	0.9	89–91	62.4	60.5
Lipids (%)	0.5–3	0.12	0.6	2.2	10–29
Protein (%)	4–12	0.93	0.4	16.2–18.6	13.6–16.9
Protein energy value (%)	9.4	1.9	0.8	18.8	11.1
**Micronutrients**					
Ash (%)	3–10	0.43	0.5–1	5–7	1.3–1.6
Magnesium (mg/100 g)	20.8–420	88	49	2002	220.1
Sodium (mg/100 g)	100–266.6	-	-	5.32	20.1
Potassium (mg/100 g)	2284–2460	1282	11	4977	882.4
Calcium (mg/100 g)	54.8–554	370	190	584	34.9
Iron (mg/100 g)	4.3–15	30.2	3.3	41.8	4.6
Vitamin C (mg/100 g)	69.8	-	-	110	-
**Bioactive Compounds**					
Tannins (%)	1.8–9.3	-	-	0.02	0.02
Caffeine (%)	1.2	-	0.1	1.4	0.4
CGAs (%)	10.7–12.6	-	-	15.8	11.5
Melanoidins (%)	15	-	-	17–23	13–25
**References**	[11,12,13,14,15]	[14,16,17]	[14,18,19,20,21]	[11,22,23,24]	[11,25,26,27]

**Table 2:** Nutrition claims and health-promoting properties of coffee by-products associated with their composition that is shown in Table 1.

By-Product	Nutrition Claims ^1^	Health-Promoting Properties
Cascara	High in fiber ^2^ Low in fat ^3^ Source of potassium, calcium, magnesium and vitamin C ^4^	Anti-diabetic [28,29] Antioxidant [13,30,31] Anti-inflammatory [32,33]
Mucilage	Low in fat Source of potassium, calcium and magnesium	Antioxidant [34]
Parchment	High in fiber Low in fat Source of calcium and magnesium	Hypoglycemic [19] Hypolipidemic [19]
Silverskin	High in fiber Low in fat Source of proteins ^5^ Source of potassium, magnesium, calcium and vitamin C	Prebiotic [25,35] Anti-obesity [36] Anti-diabetic [37,38,39] Antioxidant [39,40,41,42,43] Anti-inflammatory [33,44] Skin health [44,45,46,47]
Spent coffee grounds	High in fiber Source of proteins Source of potassium and magnesium	Prebiotic [25] Anti-diabetic [48] Antioxidant [1,25,49,50] Anti-inflammatory [51]

^1^ “Any claim which states, suggests or implies that a food has particular beneficial nutritional properties due to the energy (calorific value) it provides or does not provide; or to the nutrients or other substances it contains or does not contain” [52]. ^2^ A claim that a food is high in fiber may only be made where the product contains at least 6 g of fiber per 100 g. ^3^ A claim that a food is low in fat may only be made where the product contains no more than 3 g of fat per 100 g for solids. ^4^ A claim that a food is a source of vitamins and/or minerals may only be made where the product contains at least 15% of the recommended allowance per 100 g of product. (Recommended daily allowances (RDAs) for potassium, magnesium, calcium and vitamin C are 3.5 g, 300 mg, 800 mg and 60 mg, respectively) (See Table S1). ^5^ A claim that a food is a source of protein may only be made where at least 12% of the energy value of the food is provided by protein [53].

## Supplementary Materials

The following are available online at https://www.mdpi.com/2218-273X/10/9/1219/s1, Table S1: Micronutrient recommended daily allowances (RDAs) and 15% of the RDAs.
