# Supplementary material for: Applications of Compounds from Coffee Processing By-Products
Source: Biomolecules. 2020 Aug 21;10(9):1219. doi: 10.3390/biom10091219 (PMC7564712; doi:10.3390/biom10091219)
Supplement: Supplementary file 1 [file biomolecules-10-01219-s001.pdf]

Table S1. Micronutrient recommended daily allowances (RDAs) and 15% of the RDAs.

Micronutrient	RDAs[1]	15 % RDAs
Potassium	3.5 g	0.52 g
Magnesium	300 mg	45 mg
Calcium	800 mg	120 mg
Vitamin C	60 mg	9 mg

[1] According to the European Council (90/496/EEC). *Off. J. Eur. Union* **1990**, 33, 18–63.
